# Clinical characteristics and treatment outcomes of kidney transplant recipients with *de novo* urothelial carcinoma: thirty years of experience from a single center

**DOI:** 10.1186/s12894-023-01232-7

**Published:** 2023-04-28

**Authors:** Chunkai Du, Mengmeng Zheng, Zhipeng Wang, Jian Zhang, Jun Lin, Lei Zhang, Ye Tian, Yichen Zhu

**Affiliations:** Department of Urology, Beijing Friendship Hospital, Capital Medical University, No. 95 Yong’an Road, Xicheng District, Beijing, 100050 China

**Keywords:** Kidney transplantation, Urothelial carcinoma, Cisplatin-based chemotherapy, Bladder infusion chemotherapy, Rapamycin, Clinical prognosis

## Abstract

**Background:**

*De novo* urothelial carcinoma (UC) is a leading cause of death after kidney transplant (KT). The efficacy of various treatments, apart from surgery, and the prognosis for patients with urothelial carcinoma after kidney transplantation remain unclear.

**Methods:**

We retrospectively reviewed the efficacy of chemotherapy with gemcitabine + cisplatin (GC) or gemcitabine + carboplatin (GCa), bladder infusion chemotherapy, and immunosuppression therapy for *de novo* UC in kidney transplantation recipients at different sites and T stages. We evaluated the prognosis and compared the difference using Kaplan-Meier analysis and the log-rank test.

**Results:**

Of the 97 kidney transplantation recipients with *de novo* UC, 51 (52.6%) were diagnosed with upper urinary tract carcinoma (UTUC), 17 (17.5%) with bladder carcinoma (BC), and 29 (29.9%) with both UTUC and BC. The five-year survival rates for BC, UTUC, and BC + UTUC with ≤ T1 stage were 100%, 88.2%, and 57.7%, respectively, while the survival rates for UTUC, BC + UTUC with ≥ T2 stage were 90.2% and 48.2%. Cyclosporine A significantly improved progression-free survival (PFS) in UTUC with ≤ T1 stage (*p* = 0.017). Rapamycin significantly improved PFS in UTUC with ≥ T2 stage (*p* = 0.026). Bladder infusion chemotherapy and GC/GCa chemotherapy had no significant effect on each T stage and site. Patients with UTUC + BC had the poorest overall survival (OS) compared with those with BC and UTUC.

**Conclusion:**

The prognosis of UC in different sites varies. GC/GCa chemotherapy and bladder infusion chemotherapy appear to have no effect on prognosis. Rapamycin can delay the progression of advanced UTUC.

## Introduction

De novo urothelial carcinoma (UC) is a major cause of death after kidney transplant (KT), especially in East Asia, where it is believed to be related to aristolochic acid [1, 2]. KT recipients are at a three-fold greater risk for UC compared to the immunocompetent population [3, 4], and in the East this ratio can be as high as 14 times [5]. In our center, a previous study found the incidence of uroepithelial carcinoma after kidney transplantation to be 1.04% [6]. The multiple occurrences, tendency towards high grade, and advanced stage of de novo UC contribute to the substantial mortality rate of KT recipients [7]. However, the lack of relevant studies has prevented the development of a consensus on an appropriate management strategy. In this study, we review the clinical characteristics and outcomes of 97 KT recipients with de novo UC to determine the optimal treatment regimen.

## Methods and materials

### Study approval and patient consent

The protocol for this research project has been approved by a suitably constituted Ethics Committee of the institution and it conforms to the provisions of the Declaration of Helsinki. Committee of Beijing Friendship Hospital, Approval No. YYYXYJ-2021-335. The requirement for informed patient consent was waived due to the retrospective nature of this study.

### Study cohort

The cohort of this cross-sectional, single-center, retrospective study included 97 RT recipients diagnosed with *de novo* UC who underwent radical surgery at Beijing Friendship Hospital from January 1992 to December 2021. Patients with a history of secondary transplantation, radiotherapy, any other tumor before UC, and renal allograft loss before a diagnosis of UC were excluded from analysis.

### Definitions

Overall survival (OS) was defined as the time to patient death, relapse-free survival (RFS) as the time to recurrence at any site, and progression-free survival (PFS) as the time to metastasis to any other site.

### Clinical data

We collected patient demographics and clinical characteristics from the electronic database and paper medical records of Beijing Friendship Hospital. The variables included sex, age at RT, type and duration of dialysis, immunosuppression regimens, age at UC diagnosis, post-transplant duration, disease stage, general tumor characteristics, oncologic outcome, history of aristolochic acid (AA) exposure, and use of prophylactic nephrectomy, bladder infusion chemotherapy, and/or adjuvant chemotherapy with gemcitabine + cisplatin (GC) or gemcitabine + carboplatin (GCa). We set the cut-off dates for OS, PFS, and RFS as December 31, 2021.

### Chemotherapy regimens

Gemcitabine (800 mg/m^2^) was intravenously infused within 30 min on days 1, 8, and 15, while cisplatin (70 mg/m^2^) was administered within 2 h on day 2. For patients with impaired renal function, cisplatin was replaced with carboplatin (area under the curve = 5). Each chemotherapy cycle was 4 weeks. Patients with severe (grade ≥ 2) marrow suppression received supportive therapy with hematopoietic growth factor. If the patient did not meet the eligibility criteria, the chemotherapy cycle was postponed until recovery or discontinued because of disease progression or unacceptable toxicity.

### Statistical analysis

Categorical variables were compared using the Fisher’s exact test or chi-square test and are presented as numbers and percentages. Continuous variables with normal distributions were compared with the *t*-test and are presented as the mean ± standard deviation, while continuous variables with non-normal distributions are presented as the median and interquartile range. The Kolmogorov-Smirnov test was used to assess the normality of the distribution of continuous variables. Kaplan-Meier analysis and the log-rank test were used to evaluate OS, RFS, and PFS. For evaluation of RFS and PFS, the chemotherapy and infusion groups were limited to patients who received treatment upon a confirmed diagnosis of UC, while all others were included in the control group. All statistical analyses were performed using IBM SPSS Statistics for Windows, version 26.0 (IBM Corporation, Armonk, NY, USA). A two-tailed probability (*p*) value of < 0.05 was considered statistically significant.

## Results

### Patient demographics and clinical characteristics

As shown in Table 1, the average time from KT to diagnosis of de novo UC was 98.1 ± 66.4 months. Of the 97 recipients of a renal transplant who were diagnosed with de novo UC, 77 (79.4%) were female and 20 (20.6%) were male. Among them, 82 (84.5%) received hemodialysis before KT (mean duration 12.8 ± 16.7 months), 65 (81.3%) had a confirmed history of exposure to AA, and 51 (52.6%) were diagnosed with upper urinary tract carcinoma (UTUC), 17 (17.5%) with bladder carcinoma (BC), and 29 (29.9%) with both UTUC and BC. The p values for the different groups are shown in Table 2.

**Table 1:** The characteristic of all cases

		bladder	upper tract	bladder + upper tract	total
count		17	51	29	97
sex					
	male	6	14	4	20
	female	11	41	25	77
dialysis type					
	hemodialysis	12	44	26	82
	Peritoneal Dialysis	1	1	0	2
	none	0	1	1	2
	hemodialysis + peritoneal dialysis	1	0	0	1
Left-right					
	left	-	28	12	40
	right	-	22	13	35
	both	-	1	4	5
T					
	0	0	2	0	2
	1	14	25	14	53
	2	2	14	9	25
	3	1	9	5	15
	4	0	1	1	2
tumor grand					
	1	9	15	0	24
	2	2	24	16	42
	3	6	12	13	31
multiple occur					
	yes	9	32	-	41
	no	8	18	-	26
time between transplantation and tumor (month)		74.8 ± 82.3	114.3 ± 61.3	83.2 ± 59.0	98.1 ± 66.4
age at transplantation (year)		51.7 ± 11.6	46.8 ± 8.3	49.1 ± 8.8	48.3 ± 9.2
dialysis time (month)		23.1 ± 32.6	9.7 ± 7.8	12.3 ± 13.0	12.8 ± 16.7

*The total number of some items are not 97 due to some data loss.

**Table 2:** The survival time in each part and T stage

		Overall Survival time (month)	Relapse-free Survival (month)	Progression-free Survival (month)
Bladder				
	T1 and lower stage	147.86 ± 15.63 (95%CI: 117.22–178.50)	21.28 ± 9.35 (95%CI: 2.95–39.61)	43.39 ± 13.67 (95%CI: 16.69–70.28)
Upper-Tract				
	T1 and lower stage	145.92 ± 12.60 (95%CI: 121.22-170.62)	-	90.40 ± 14.21 (95%CI: 62.55-118.25)
	T2 and higher stage	168.06 ± 15.30 (95%CI: 138.07-198.05)	-	108.23 ± 20.63 (95%CI: 67.81-148.65)
Upper-Tract and Bladder				
	T1 and lower stage	112.92 ± 27.92 (95%CI:58.21-167.64)	26.86 ± 4.81 (95%CI:17.42–36.29)	48.92 ± 8.88 (95%CI:31.52–66.32)
	T2 and higher stage	79.62 ± 22.00 (95%CI:36.51-122.73)	49.07 ± 18.21 (95%CI:13.38–84.77)	171.776 ± 19.12 (95%CI:134.29-209.23)

### BC

#### Stage ≤ T1

A total of 14 patients were diagnosed with stage ≤ T1 BC and underwent TURBT. One patient received bilateral nephroureterectomy and cystectomy after TURBT. Table 3 shows the OS, RFS, and PFS rates of these patients. The 5-year OS rate was 100%. Of the 14 patients, 8 (57.1%) received bladder infusion chemotherapy, and one received it for BC recurrence. The OS rates of the two groups were 100%, and there was no significant difference in RFS and PFS rates between them (p = 0.071 and 0.400, respectively). In total, 7 (50.0%) patients were treated with cyclosporine A (CsA) and 6 (42.9%) with tacrolimus (TAC), and there were no significant differences in OS, RFS, and PFS rates between the CsA and TAC groups (p = 0.281, 0.755, and 0.937, respectively).

**Table 3:** The P value in different group

		Rapamycin			Bladder infusion Chemotherapy			GC/GCa chemotherapy			CNI drugs		
		OS	RFS	PFS	OS	RFS	PFS	OS	RFS	PFS	OS	RFS	PFS
BC													
	T1 and lower stage	-	-	-	-	0.071	0.4	-	-	-	0.281	0.755	0.937
UTUC													
	T1 and lower stage	0.057	-	0.122	0.343	-	0.199	-	-	-	0.187	-	0.017
	T2 and higher stage	0.317	-	0.026	-	-	-	0.132	-	0.521	-	-	-
BC + UTUC													
	T1 and lower stage	0.225	0.98	0.274	0.665	0.778	0.228	-	-	-	0.672	0.361	0.895
	T2 and higher stage	0.602	0.362	0.436	0.284	0.697	0.825	0.507	0.885	0.436	-	-	-

BC: Bladder Carcinoma; UTUC: Upper-tract Urocilial Carcinoma

#### Stage ≥ T2

Only three patients were diagnosed with stage ≥ T2 BC. Two patients with stage T2 BC received TURBT, and one with stage T3 BC underwent partial cystectomy without infusion or GC/GCa chemotherapy. Of the two patients with stage T2 BC, one received infusion chemotherapy, and the other received GC/GCa chemotherapy. Two patients died after OS durations of 122 and 142 months, while the third was still alive after 48 months. All patients experienced disease recurrence with RFS durations of 40, 3, and 18 months, respectively. All patients developed metastasis to the upper urinary tract with PFS durations of 82, 9, and 23 months, respectively.

### UTUC

#### Stage ≤ T1

**Fig. 1:**
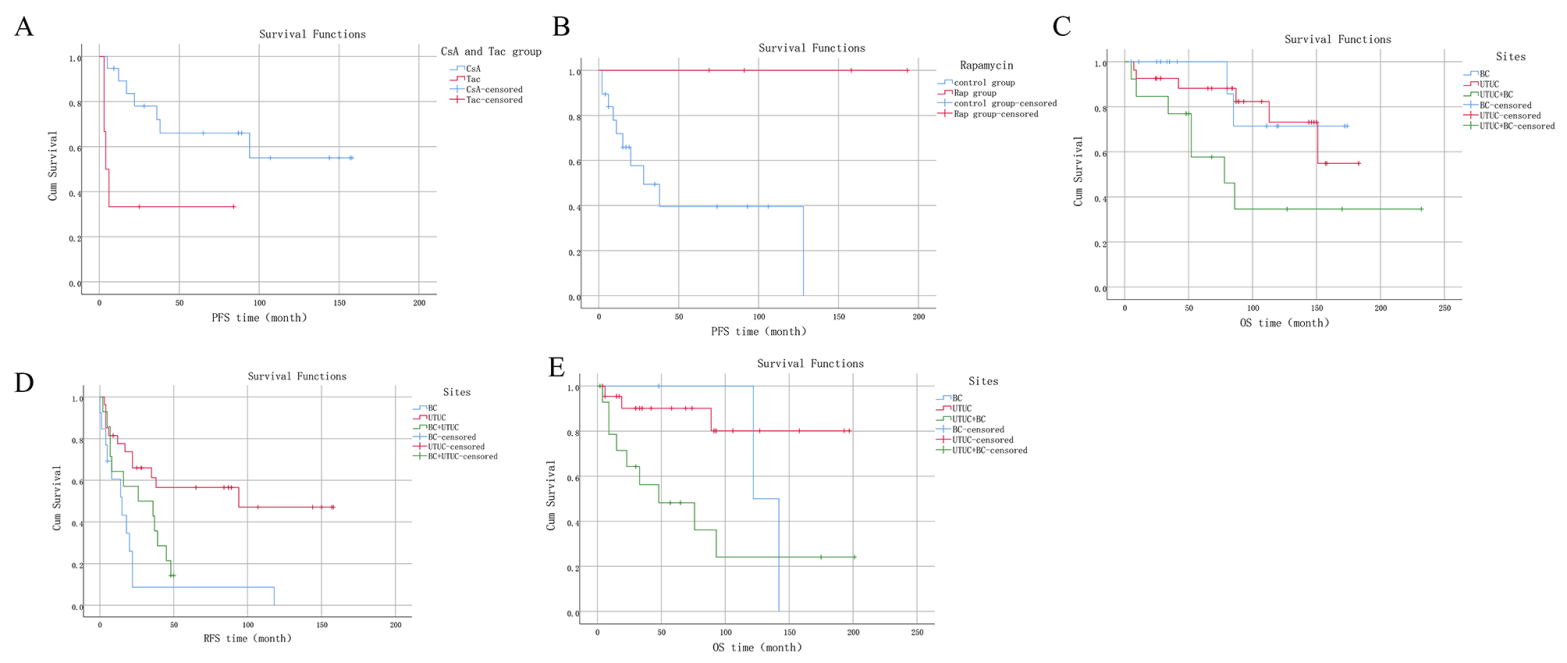
The survival of the patients in different group A.The PFS time between CsA group and Tac group in T1 and lower stage of UTUC; B. The PFS time between Rap group and control group in T2 and higher stage of UTUC; C. The OS time between different sites in T1 and lower stage; D. The RFS time between different sites in T1 and lower stage; E. The OS time between different sites in T2 and higher stage

A total of 27 patients diagnosed with stage ≤ T1 UTUC underwent nephroureterectomy without receiving GC/GCa chemotherapy. Table 3 shows their OS and PFS rates. The 5-year OS rate for these patients was 88.2%. After the initial diagnosis, 5 patients (18.5%) and 4 (14.8%) after recurrence had their immunosuppression regimen changed to rapamycin (RAP), while 18 (66.7%) did not receive RAP. There was no significant difference in OS and PFS rates between these patients (p = 0.057, 0.122). Of these patients, 19 (70.4%) were treated with CsA and 6 (22.2%) with TAC, with PFS being significantly longer in the CsA group than in the TAC group (p = 0.017) (Fig. 1A). However, there was no significant difference in OS rates between the two groups (p = 0.184). Among these 27 patients, 8 (29.6%) received infusion chemotherapy for BC after the initial diagnosis, 2 (7.4%) after recurrence, and 17 (63.0%) did not receive infusion chemotherapy, with no significant difference in clinical prognosis observed between the groups (p = 0.343 and 0.199) for OS and PFS, respectively.

#### Stage ≥ T2

A total of 24 patients with stage ≥ T2 UTUC underwent nephroureterectomy. Table 3 shows the OS and PFS rates of these patients. The 5-year OS rate was 90.2%. Out of the 24 patients, 5 (20.8%) received GC/GCa chemotherapy while 19 (79.2%) did not. There was no significant difference in the OS and PFS rates between the two groups (p = 0.132 and 0.521, respectively). The immunosuppression regimen was changed to RAP for 4 (16.7%) patients after the initial diagnosis, but there was no significant difference in OS between patients treated with and without RAP (p = 0.317). PFS was superior in the RAP group compared to the control group (p = 0.026) (Fig. 1B).

### UTUC combined with BC

#### Stage ≤ T1

A total of 14 patients diagnosed with stage ≤ T1 UTUC + BC underwent nephroureterectomy, TURBT or partial cystectomy, with only one patient receiving GC/GCa chemotherapy. Table 3 shows the OS, RFS, and PFS rates of these patients, with a 5-year OS rate of 57.7%. The immunosuppression regimen was changed to RAP for 2 (14.3%) patients after the initial diagnosis and 1 (7.1%) after recurrence, while 11 (78.5%) did not receive RAP. No significant differences in clinical prognosis were found between patients treated with or without RAP (p = 0.225, 0.980, and 0.274, respectively). Bladder infusion chemotherapy was administered to 10 (71.4%) patients after the initial diagnosis and 1 (7.1%) after recurrence, while 3 (21.4%) did not receive infusion chemotherapy. No significant differences in clinical prognosis were found among these three groups (p = 0.665, 0.778, and 0.288, respectively). However, there was no significant difference in OS, RFS, and PFS rates between 10 (71.4%) patients treated with CsA and 2 (14.3%) treated with TAC (p = 0.672, 0.361, and 0.895, respectively).

#### Stage ≥ T2

A total of 15 patients diagnosed with stage ≥ T2 UTUC + BC underwent nephroureterectomy, TURBT, partial cystectomy, or cystectomy, with a 5-year OS rate of 48.2%. Only 3 (20.0%) patients had their immunosuppression regimen changed to RAP, and there was no significant difference in OS, RFS, and PFS rates between patients treated with or without RAP (p = 0.602, 0.362, and 0.436, respectively). Of the 15 patients, only 6 (40.0%) received bladder infusion chemotherapy, and no differences in OS, RFS, and PFS rates were found between the two groups (p = 0.284, 0.697, and 0.825, respectively). No significant differences in OS, RFS, and PFS rates were found between 4 (26.7%) patients who received GC/GCa chemotherapy and 11 who did not (p = 0.507, 0.885, and 0.436, respectively).

### Prognosis according to site and T stage

#### Site

Patients were classified into the BC, UTUC, or UTUC + BC group based on the primary tumor site. For stage ≤ T1 disease, there were significant differences in OS and RFS among the three groups (p = 0.045 and 0.001, respectively) (Fig. 1C and D). OS was significantly reduced in the UTUC + BC group compared to the BC and UTUC groups, while the UTUC group had the longest RFS and the BC group had the shortest. Meanwhile, there were no significant differences in PFS among the three groups (p = 0.183). For stage ≥ T2, there was a significant difference in OS among the three groups (p = 0.017) (Fig. 1E). OS was comparatively shorter in the UTUC + BC group than in the UTUC group. Notably, there were no significant differences in RFS and PFS among the three groups (p = 0.180 and 0.078, respectively).

#### T stage

Comparisons of patient prognosis based on T stage revealed no significant difference in survival.

## Discussion

UC is a common long-term complication following KT, which severely threatens survival. The incidence of UC is approximately ten times greater in KT recipients than in the general population, particularly in East Asia [3]. This may be due to the use of herbal medicines containing aristolochic acid (AA), which is nephrotoxic and has been linked to kidney failure and urothelial malignancy [8]. Exposure to AA is associated with an increased incidence of upper tract UC [9], which is consistent with the patient cohort included in this study. Previous studies of UC after KT have generally been small and lacked long-term follow-ups to assess patient prognosis. In this study, medical records of 97 patients were reviewed, which included follow-up periods of more than 20 years. This study is thus among the largest to date and included a relatively long follow-up period.

Most UC after KT originates in the recipient’s urinary system. Although rare, most malignancies in transplanted kidneys have been confirmed to originate from the donor. There are two main reasons for this phenomenon: exposure to AA and immunity-related factors. AA exposure can cause permanent mutations to the TP53 gene, even after relatively long periods of exposure [1, 8, 10]. KT recipients with a history of AA exposure can develop gene mutations not associated with the transplanted kidney. Although the immune function of the recipient is inhibited by immunosuppressive drugs, attacks on the transplanted kidney can still occur.

This study looked at how the location and size of tumors affects patient outcomes. For tumors in the same location, the size of the tumor did not affect patient survival, recurrence, or progression of the disease. However, tumors with the same size could have different characteristics. For example, patients with bladder cancer tended to survive longer if they were diagnosed early, but were more likely to have the cancer come back. Patients with both bladder cancer and upper tract urothelial carcinoma tended to have shorter survival rates. Patients diagnosed with late-stage bladder cancer had shorter survival rates than those diagnosed with early-stage bladder cancer. However, the study had a small sample size, so these results may not be accurate. There was not much difference in the time it took for the cancer to come back or progress in the three groups. Bladder cancer is usually diagnosed early because it causes hematuria, or blood in the urine, which is an obvious symptom. However, patients with bladder cancer are more likely to have the cancer come back, so they should have regular cystoscopies. Patients with both bladder cancer and upper tract urothelial carcinoma had the worst prognosis.

RAP, an mTOR inhibitor, is an immunosuppressant that has garnered increased attention due to its potential antitumor effects, specifically in inhibiting tumorigenesis and progression through the PI3K-AKT pathway [11]. However, the evidence for its antitumor effects is still insufficient and controversial, with most studies done in vitro or small-scale in vivo. Large-scale clinical studies are needed for confirmation of its effectiveness. In this study, RAP significantly delayed the recurrence and progression of stage ≥ T2 UTUC, indicating some inhibitory effect on tumor progression. More cases are needed to analyze this effect specifically by factors such as duration and in vivo concentration.

For advanced UC, platinum-based chemotherapy is the first-line treatment option [12, 13], but it is rarely used in KT recipients due to platinum’s nephrotoxicity. The safety of platinum-based chemotherapy for KT recipients has been demonstrated in previous studies [14], but its effect on the prognosis of patients with stage ≥ T2 disease remains unclear. In this study, platinum-based chemotherapy had no significant effect on the prognosis of patients with stage ≥ T2 disease. The effect of the GC/GCa regimen for distant metastases in KT recipients also remains unknown.

Bladder infusion chemotherapy is recognized for reducing the rate of tumor recurrence in non-muscle invasive bladder cancer and reducing the risk of upper tract urothelial carcinoma recurrence [15, 16]. However, its use in the renal transplant population remains controversial due to concerns about the safety of using BCG in an immunosuppressed state [17]. Although some studies have shown that bladder infusion chemotherapy with BCG is both safe and effective [18, 19], there have been cases of systemic infection following bladder infusion with BCG [20]. In this study, we used non-live vaccine-like drugs, such as epirubicin and mitomycin, for bladder instillation. Bladder infusion chemotherapy was mostly reserved for patients with non-invasive bladder cancer with or without upper tract urothelial carcinoma. However, bladder infusion chemotherapy did not benefit tumor recurrence and metastasis, which may have been influenced by the low number of patients with bladder cancer and the rapid progression of upper tract urothelial carcinoma combined with bladder cancer.

Immunotherapy is not commonly used for KT recipients. The principle of immunotherapy is to improve immune function to kill tumor cells. However, in the KT population, increased immunity may raise the risk of renal allograft rejection. In one study, PD-1 was found to cause kidney transplant rejection in some patients and tumor progression in most patients [21]. Therefore, we consider the use of PD-1 or PD-L1 in the renal transplantation population to be riskier. At our center, no kidney transplant patients have been treated with PD-1 or PD-L1.

For this retrospective study, data were collected from medical records and telephone interviews. However, the survival rate may have been inflated due to missing or out-of-contact patients, leading to cases with missing information, especially survival data, being excluded from the analysis.

In conclusion, the prognosis for UTUC combined with BC is extremely poor. RAP has been shown to effectively improve patient prognosis. Prophylactic resection is recommended for UTUC combined with BC, whereas the GC/GCa regimen and bladder infusion chemotherapy have little effect on prognosis according to this study.

## Data Availability

The datasets used and/or analysed during the current study are available from the corresponding author on reasonable request.
